# Mutational Profile of Metastatic Breast Cancer Tissue in Patients Treated with Exemestane Plus Everolimus

**DOI:** 10.1155/2018/3756981

**Published:** 2018-07-24

**Authors:** Claudia Omarini, Maria Elisabetta Filieri, Stefania Bettelli, Samantha Manfredini, Shaniko Kaleci, Cecilia Caprera, Cecilia Nasso, Monica Barbolini, Giorgia Guaitoli, Luca Moscetti, Antonino Maiorana, Pier Franco Conte, Stefano Cascinu, Federico Piacentini

**Affiliations:** ^1^Division of Medical Oncology, Department of Medical and Surgical Sciences for Children & Adults, University Hospital of Modena, Italy; ^2^Division of Pathological Anatomy, Department of Diagnostic, Clinical Medicine and Public Health, University Hospital of Modena, Italy; ^3^Department of Surgery, Oncology, and Gastroenterology, University of Padova, Italy

## Abstract

**Background:**

Everolimus has been shown to overcome endocrine resistance in hormone receptor positive advanced breast cancer patients. Predictive biomarkers of everolimus efficacy have been investigated in primary breast cancer tissue without finding univocal results. The goal of this study was to investigate the mutational burden in the metastatic site of endocrine-resistant tumors treated with everolimus plus exemestane.

**Patients and Methods:**

Mass Array Sequenom platform was used to analyse genetic status of 18 cancer-related genes in 25 archival tumor specimens from metastatic lesions and available primary matched breast cancer tissue of patients treated with everolimus and exemestane for advanced disease. An exploratory analysis of everolimus efficacy in terms of progression free survival benefit and single gene mutation was carried out.

**Results:**

The overall detection rate of mutation was 30% and 38% from metastatic and primary breast cancer samples, respectively. AKT1^E17K^ was the most frequent mutated gene. No primary breast cancer and matched relapse maintained the same mutation profile. Considering molecular pathways, the most of the genes belong to PI3K pathway (AKT1^E17K^, PI3KCA^E545K^, and KIT^G565R,S709F^). In patients with detected mutations in breast and/or recurrence tissue the median PFS was 5,6 months while in the subgroup of patients with no mutations the median PFS was 7,5 months.

**Conclusions:**

The mutational status of breast cancer recurrence allows the identification of some genes potentially correlating tumor response/resistance to everolimus. The most frequently mutated genes were involved in the PI3K/AKT/mTOR pathway highlighting that the deregulation of this pathway in the relapse plays a crucial role in the mechanisms of everolimus resistance/sensitivity. Owing to the small sample size and the retrospective nature of the study, these correlations need to be validated in a large prospective study.

## 1. Introduction

Breast cancer (BC) is the most common malignant tumor in women. More than 70% of BCs are hormone receptor positive (HR+) and human epidermal growth factor receptor 2 negative (HER2-) and potentially benefit from endocrine therapies [1]. However, approximately 25% of these tumors fail to respond to hormonal treatment because of the novo or acquired resistance [2]. In the last decade, new targeted therapies have been investigated, with the aim of improving treatment efficacy in patients progressed on endocrine therapy. The double-blinded randomized placebo control, phase III BOLERO-2 study, demonstrated that everolimus plus exemestane improved progression free survival (PFS) compared with exemestane alone in HR+ HER2- advanced BC that recurred or progressed during/after nonsteroidal aromatase inhibitors [3].

Actually, the main challenge is the identification of biomarkers able to predict which patients can benefit from the addiction of targeted agents (such as everolimus) to hormonal treatment. A large number of preclinical and clinical studies tried to identify predictive biomarkers of everolimus sensitivity in endocrine resistance population. Several preclinical analyses have suggested how the presence of mutation in PI3KCA/AKT/mTOR (*phosphatidylinositol 3-kinase/protein kinase B/mammalian target of rapamycin*) pathway as well as PTEN (*phosphatase and tensin homolog*) gene loss detected on primary BC tissue correlates with everolimus benefit [4, 5]. However, the following clinical trials in BC patients did not confirm the correlation between PI3KCA/PTEN status and clinical response [6–8]. This discordance could be in part justified by the different mutational status between primary tumors and metastatic site. To the best of our knowledge, there are no data regarding the gene mutation status in metastatic tissue and tumor sensitivity to everolimus.

The main purpose of this study was to investigate the mutational burden in the metastatic site of endocrine-resistant patients treated with everolimus plus exemestane in our Institution. We used a panel of 18 genes known to be involved in the mechanism of endocrine and targeted treatments resistance. Moreover, we analysed the gene mutation status of the available matched primary tumors.

## 2. Materials and Methods

### 2.1. Patient Population and Samples

**Figure 1:**
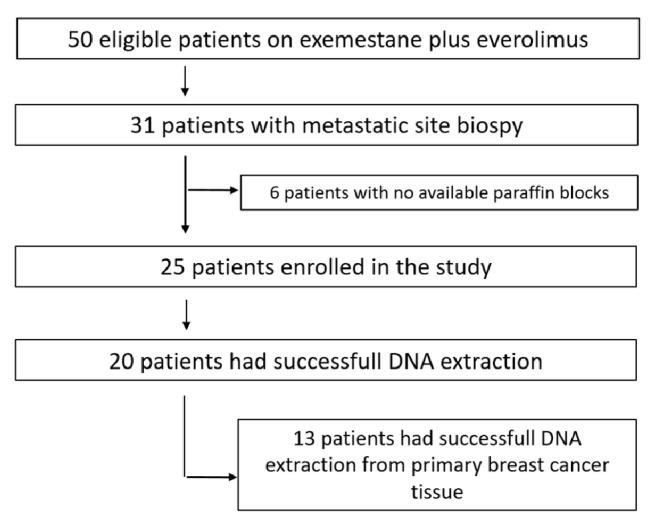
Flowchart of the study population.

We retrospectively identified fifty patients with HR+ HER2- metastatic BC progressed on/after endocrine therapies and treated with exemestane plus everolimus in routine clinical practice at the Modena Cancer Centre. All these patients wrote an informed consent to study enrolment. Clinical and pathological characteristics were collected from informatic archives. PFS on everolimus and exemestane was defined as the time elapsed between treatment initiation and first documented progression disease/death. Thirty-one patients performed a biopsy of the local or distant recurrence; twenty-five of these patients had stored paraffin blocks of metastatic tissue suitable for gene analysis. In nineteen cases, the matched primary BC was available too (Figure 1). Metastatic site biopsy was performed before starting the combination treatment in all the patients.

### 2.2. Gene Analysis

Gene analysis was performed in Molecular Biology Laboratory of Modena Pathology Department. DNA was extracted from formalin-fixed and paraffin-embedded (FFPE) metastatic and primary BC tissues. The FFPE tissues were cut and stained with haematoxylin and eosin and a pathologist selected sections containing more than 30 % tumor cells. Genomic DNA was isolated from unstained 10 *μ*m-sections using QIAamp DNA Mini kit (QIAGEN). The DNA concentration in the samples was quantified using a spectrophotometer TrineanXpose and they were diluted to a final concentration of 20 ng/*μ*l. DNA samples were analysed by OncoCarta v2.0 panel using Mass Array Sequenom platform following the manufacturer's protocols (*http://agenabio.com*). This panel is able to detect 152 somatic mutations across 18 oncogenes and tumor suppressors (AKT1, BRAF, CTNNB1, FBX4, FBXW7, FGFR2, FGFR3, GNAQ, KIT, KRAS, MAP2K1, MAP2K2, NRAS, PDGFR*α*, PIK3CA, PTPN11, SOS1, and TP53). The panel consists of 12 multiplexed wells that are run on each sample using 40 ng of input DNA from FFPE tissue. The polymerase chain reaction amplification and primer extension were performed using the OncoCarta Panel v2.0 reagents. The detection and quantify mutation frequencies are ≥ 10%. Twenty patients had successful DNA extraction from the metastatic site useful for gene analysis. In thirteen cases, we had successful DNA extraction from the matched primary BCs too.

## 3. Results

### 3.1. Study Population

Twenty-five patients have been enrolled in the study. Four patients were diagnosed with de novo metastatic BC, while all the other cases relapsed in spite of adjuvant treatments. All the patients progressed on/after aromatase inhibitors and received at least two previous lines of (chemo- or hormonal-) therapies for advanced disease. The median age at the time to start everolimus plus exemestane was 54 years (range 50-67). Regarding the tumor burden at the beginning of everolimus and exemestane, four patients had only bone disease and two patients only locoregional recurrence (chest wall and/or lymph nodes), while all other patients presented visceral disease (liver and/or lung). Tumors characteristics of both metastatic site and primary BC are reported in Table 1. According to 13th St Gallen International Breast Cancer Conference classification, seventeen patients relapsed as luminal B-like disease (HR+, HER2-, Mib1 ≥ 20%), and eight were luminal A-like BCs (HR+, Mib1 <20%). Primary BC was luminal B-like in twelve patients, whereas luminal A-like in the other ones. Of note, in two patients the BC subtypes changed from luminal A-like to luminal B-like from primary breast disease to relapse, whereas in other two cases it changed from luminal B-like to luminal A-like from breast sample to metastasis. At the time of analysis, all the patients had progressed on everolimus and exemestane. Median PFS was 6,6 months (range from 1 to 17 months).

### 3.2. Gene Mutations on Metastatic Site

Among twenty-five patients enrolled, twenty had successful DNA extraction from FFPE metastatic site. In six of these patients, the biopsy was performed on the local/regional recurrence site (breast, chest wall, or lymph nodes). In nine cases, the biopsy was performed on liver recurrence, in three patients on lung metastasis, and in two cases on the spinal bone lesions.

**Figure 2:**
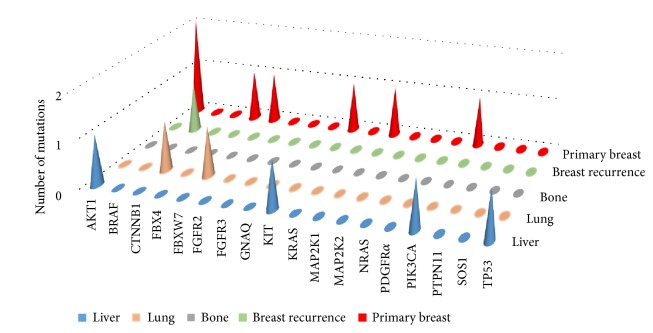
Oncogene mutations across metastatic and primary breast cancer site.

In all but one case the mutations were detected in the visceral recurrence (liver or lung) (Figure 2). Gene mutations were identified in 6 out of 20 patients analysed (30%). All but one of them had only one mutated gene (BRAF, CTNNB1, FBXW7, KIT, or PT53). Only* patient 6* presented two mutations: one on PI3KCA and one on AKT1, respectively (Table 2). Considering molecular pathways, the most of the detected genes belong to PI3K pathway (AKT1, KIT, PI3KCA, and PT53), APC pathway (CTNNB1, FBXW7, and PT53), and MAP and RAS pathways (AKT1 and KIT; AKT1 and BRAF, respectively).

### 3.3. Gene Mutations on Primary Breast Cancer

Primary BC tissue was available in nineteen patients but only in thirteen cases the DNA was successfully extracted. The overall detection rate of mutations in the primary BC was 38% (5 out of 13 patients). A single gene mutation was identified in four out of thirteen patients analysed. Only* patient 7 *presented three mutations in three different genes: FBX4, PI3KCA, and KIT. Two patients presented the same mutation on AKT1: E17K. Other two cases had a single mutation on MAP2K1 and FBXW7, respectively. Considering molecular pathways, the most of the mutated genes belong to PI3K pathway (AKT1, KIT, and PI3KCA), APC pathway (FBXW7and FBX4), and MAP and RAS pathways (AKT1 and MAP2K1; AKT1 and KIT, respectively) (Table 2).

### 3.4. Correlation between Gene Mutations on Metastatic and Primary Breast Cancer

The metastatic and the matched primary BC tissues were profiled in eight patients; in three of them no mutation has been detected in both samples. No primary BC and matched relapse maintained the same mutation profile. Two patients acquired AKT1^E17K^ mutation in the metastatic sites, while two patients lost the mutation detected in the primary tissue. In* patient 18 *a single mutation on a different gene was detected in primary tissue (FBXW7^R479Q^) and in the recurrence one (MAP2K1^D67N^) (Table 2).

Overall AKT1 was the most frequent mutated gene, with the same mutation E17K (one in the metastatic tissue and two in the relapse ones). Similarly, the E545K was the only mutation detected in PI3K gene. Mutations in KIT, PI3KCA, and FBXW7 were detected in both primary BC and relapse. Regarding molecular pathways, the most of the genes belong to PI3K, MAP, and APC pathways, with four mutated genes involved in each pathway.

In patients with detected mutations in breast and/or recurrence tissue, the median PFS was 5,6 months (range from 1 to 15 months), while in the subgroup of patients with no mutations the median PFS was 7,5 months (range from 2 to 17 months) (Table 3).

## 4. Discussion

Everolimus is a selective inhibitor of mTOR, a multiprotein complex controlled by mitogenic positive signal (mediated through PI3K/AKT pathway) and by negative regulators (such as PTEN) [9]. Receptors tyrosine kinase signalling and/or acquired mutation in genes involved in PI3K/AKT pathway can activate constitutively this complex [9, 10]. Hormone resistance models have shown that tumors can loss endocrine responsiveness through activation of PI3K/AKT/mTOR [11]. The combination of mTOR inhibitors such as everolimus and endocrine therapy can restore hormone sensitivity to previously resistant tumors cells [12]. Everolimus has been mainly investigated in combination with tamoxifen in TAMRAD trial and with exemestane in BOLERO2 trial [3, 13]. The combination significantly reduced the risk of disease progression and improved the clinical benefit rate compared with endocrine therapy alone in metastatic BC patients resistant to aromatase inhibitors. Translational studies of these two trials tried to find biomarkers able to predict a patient's positive response or resistance to everolimus [6, 7]. In spite of preclinical evidence showing a predictive role of specific genetic aberrations in the PI3K/AKT/mTOR pathway [14, 15], clinical data failed to confirm the correlation between a specific mutation status in the primary BC tissue and sensitivity/resistance to everolimus.

On these premises, we analysed a panel of 18 genes known to be involved in endocrine and targeted treatment resistance in metastatic BC samples. Our study is the only one that performed the gene analysis in metastatic tissue of endocrine resistance BC patients before starting everolimus. That is extremely important considering that during the natural history of the disease, metastases can acquire different biological profiles as compared to their matched primary tumor [16, 17].

In our analysis, the overall detection rate of mutations was 30%. Most of detected genes belong to PI3K pathway, supporting this pathway as a key-point in endocrine resistance BCs. The mutated genes in PI3K pathway were PI3KCA, AKT1, KIT, and PT53. All these genes are known to be involved in BC progression and metastatization [18]. Several preclinical studies have shown how PI3KCA and AKT1 mutations were able to activate PI3K pathway and to be driver of BC progression. In particular, PI3KCA is mutated and/or amplified in ~30% of BCs [19]. In the TAMRAD study 45 primary tumor samples of BC were screening for mutation status. No relationship between PI3K mutations and everolimus efficacy was found. The overall detection rate of mutations was 22%. Nine patients (5 in tamoxifen/everolimus arm and 4 in tamoxifen alone arm) had a PI3K mutation: two in the exons 9 (mutation E542K) and seven in exon 20 (mutation H1047R) [7]. Furthermore, the BOLERO-2 study failed to identify any specific gene mutation associated with a greater benefit from everolimus, in both tumor tissue and plasma cell-free DNA. Gene analyses were performed on 302 FFPE archival tumor tissue. The genes most frequently mutated were PI3KCA (47.6%), CCND1 (31.3%), TP53 (23.3%), and FGFR1 (18.1%). None of these genes such as the pathways alteration of which they were components predicted the PFS benefit with everolimus. However, quantitative differences in everolimus benefit were observed between patients subgroup defined by the exon specific mutation in PI3KCA genes. In particular, patients with the PI3KCA exon 9 (helical domain) mutation had greater PFS benefit compared with those with exon 20 (kinase domain) mutation [6]. The relationship between the PIK3CA exon 9 status and the efficacy of everolimus was investigated and confirmed in the subgroup analysis of the Phase II study of neoadjuvant everolimus plus letrozole in HR+ BC [20]. Interestingly, in our analysis* patient 6* with both PI3KCA^E545K^ (exon 9) and AKT1^E17K^ mutation experienced the greatest benefit from everolimus treatment with a PSF of 15 months.

Notably, the activating mutations in AKT gene are reported in 1.4%–8% of BCs, exclusively in HR+ tumors [21]. AKT1^E17K^ is the most commonly reported mutation. This mutation increases gene activity by promoting constitutive localization of AKT1 to the plasmatic membrane [22]. In vitro studies have shown how high levels of pAKT were predictor of sensitivity to everolimus [21]. In our series,* patient 10* and* patient 13* with AKT1^E17K^ mutation only in the primary BC had an extremely poor PFS on everolimus (5 and 2 months, respectively).

Regarding TP53, another gene involved PI3K pathway, in vitro evidence has shown how R273H mutation enhanced proliferation, invasion, and drug resistance [23]. RNA analysis confirmed how TP53^R273H^ mutants had the apoptosis pathway less active than TP53 wild type ones [23]. In accordance with this finding,* Patient9* whit TP53^R273H^ mutation detected in the metastatic liver biopsy rapidly progressed on the combination treatment (PFS = 1 month).

Less evidence is available regarding the predictive role of the other mutated genes. In our series, poor PFS were achieved by* patient 2 with *BRAF^R444W^,* patient 15* with KIT^G565R^, and* patient 18* with FBXW7^R479Q^ mutation (6, 3, and 5 months, respectively). These findings are consistent with available data in literature deriving from preclinical evidence but not specifically on BC [24–26]. Globally, median PFS in our population was higher in wild type patients compared with mutated ones. This finding is in accordance with the conclusions of the BOLERO2 investigators where patients with no alteration or a single genetic alteration received a greater PFS benefit from everolimus.

Considering the correlation between gene mutation status in the metastatic and matched BC tissue, no patient maintained the same mutation profile. In contrast with our evidence are the data from the mutational analysis performed in the metastatic tissue of 56 patients enrolled in the BOLERO2. In this subgroup analysis, the genetic profile of metastatic and primary tumors was generally similar, with a statistically increased mutation rates for ESR1, MDM2, and DNMT3A in the metastatic samples [6]. This discordance could be justified by the fact that our population was highly pretreated. In fact, it should be noted that all our patients received at least two lines of treatment for metastatic disease before starting everolimus and more than 80% of them were treated with chemotherapy for advance disease compared to 26% in BOLERO2 study. The discordance between the mutational profile of metastatic and primary BC tissue further stresses the importance of the biopsy of the metastatic site in order to catch important molecular markers that can evolve during the disease progression. Finally, the high rate of mutations detected in the primary BCs tissue (5 of 13 analysed patients, 38%) could highlight a more aggressive behaviour of these tumors and the subsequent relapse in spite of the adjuvant treatments.

Nevertheless, our study presents three major limitations. The first is the small sample size due to preanalytic bias in DNA extraction. Furthermore, a possible sampling bias must be taken into account due to the heterogeneity of the metastatic disease, where different areas of the same lesion may show different genomic profiles [27, 28]. Finally, our study focused on gene mutations, but the recent evidence underline how microRNA expression, protein expression, and abnormalities in DNA methylation may influence the response/resistance to antitumor treatments, independently from genes profile [29, 30].

## 5. Conclusion

The mutational status of BC recurrence allows the identification of some genes potentially correlating to tumor response/resistance to everolimus plus exemestane. The most frequently mutated genes among those investigated were involved in the PI3K/AKT/mTOR pathway. It is likely that the deregulation of this pathway in the relapse plays a crucial role in the mechanisms of everolimus resistance/sensitivity. Owing to the small sample size and the retrospective nature of the study, these correlations are purely exploratory and need to be validated in large prospective studies. Future research should be directed not only on the analysis of genes profile but also on the RNA expression, proteins level, and epigenetic alteration.

## Figures and Tables

**Table 1:** Tumors characteristics of metastatic site and matched primary breast cancer.

	**Relapse**		**Primary tumor**
	**Site of biopsy**	**relapse characteristics**	**Primary tumor characteristics**
Patient 1	liver	CDI ER90%, PgR 5%, Mib1 30%, HER2 1+	CDI ER100%, PgR60%, MIb1 25%, HER2 1+
Patient 2	breast	CDI ER60%, PgR 95%, Mib1 10%, HER2 1+	CDI ER98%, PgR98%, MIb1 15%, HER2 1+
Patient 3	liver	CDI ER40%, PgR30%, Mib1 40%, HER2 0	CDI ER98%, PgR70%, MIb1 30%, HER2 1+
Patient 4	liver	CDI ER100%, PgR 50%, Mib1 40%, HER2 1+	CDI ER100%, PgR80%, MIb1 25%, HER2 1+
Patient 5	chest wall	CDI ER 99%, PgR70%, Mib1 15%, HER2 0+	CDI ER 99%, PgR 70%, MIB1 15%, HER2 1+
Patient 6	liver	CDI ER 95%, PgR2%, Mib1 25%, HER2 1+	CDI ER 85%, PgR 70%, MIB1 35%, HER2 1+
Patient 7	lung	CDI ER 85%, PgR25%, Mib1 15%, HER2 1+	CDI ER 80%, PgR 60%, MIB1 25%, HER2 0+
Patient 8	breast	CDI ER 100%, PgR100%, Mib1 30%, HER2 1+	CDI ER 100%, PgR 100%, MIB1 35%, HER2 1+
Patient 9	liver	CDI ER 99%, PgR99%, Mib1 50%, HER2 1+	CDI ER 80%, PgR 60%, MIB1 25%, HER2 0+
Patient 10	liver	CDI ER 90%, PgR60%, Mib1 22%, HER2 1+	CDI ER 50%, PgR 50%, Mib1 10%, HER2 1+
Patient 11	liver	CDI ER 80%, PgR0%, Mib1 40%, HER2 1+	CDI ER 90%, PgR 50%, MIB1 25%, HER2 1+
Patient 12	skin	CDI ER 99%, PgR50%, Mib1 25%, HER2 1+	*not applicable*
Patient 13	bone	CDI ER 70%, PgR0%, Mib1 35%, HER2 0+	CDI ER 80%, PgR 60%, MIB1 25%, HER2 0+
Patient 14	bone	CDI ER 50%, PgR5%, Mib1 25%, HER2 0+	*not applicable*
Patient 15	liver	CDI ER 100%, PgR10%, Mib1 20%, HER2 1+	*not applicable*
Patient 16	lymph node	CDI ER 70%, PgR0%, Mib1 25%, HER2 1+	CDI ER 90%, PgR 80%, MIB1 8%, HER2 0+
Patient 17	liver	CDI ER 95%, PgR80%, Mib1 15%, HER2 0+	CDI ER 100%, PgR 100%, MIB1 15%, HER2 0+
Patient 18	lung	CDI ER 90%, PgR2%, Mib1 30%, HER2 1+	CDI ER 50%, PgR 65%, MIB1 25%, HER2 0+
Patient 19	lung	CDI ER 95%, PgR25%, Mib1 10%, HER2 1+	CDI ER 60%, PgR 10%, MIB1 25%, HER2 1+
Patient 20	lung	CDI ER 75%, PgR50%, Mib1 10%, HER2 1+	CDI ER 75%, PgR 20%, MIB1 15%, HER2 0+
Patient 21	bone	CDI ER 95%, PgR65%, Mib1 25%, HER2 1+	*not applicable*
Patient 22	bone	CDI ER 95%, PgR95%, Mib1 10%, HER2 1+	CDI ER 100%, PgR 85%, MIB1 3%, HER2 0+
Patient 23	liver	CDI ER 90%, PgR25%, Mib1 40%, HER2 0+	*not applicable*
Patient 24	lung	CDI ER 50%, PgR50%, Mib1 30%, HER2 1+	CDI ER 70%, PgR 50%, MIB1 25%, HER2 1+
Patient 25	lymph node	CDI ER 100%, PgR5%, Mib1 15%, HER2 0+	*not applicable*

**Table 2:** Mutations detected in relapse tissue and in primary breast cancer.

	**Relapse mutations**			**Primary tumor mutations**		
	**status**	**gene**	**mutation**	**status**	**gene**	**mutation**
Patient 1	wild type			*not available *		
Patient 2	**mutated**	**BRAF**	**R444W**	*not available *		
Patient 3	wild type			*not available *		
Patient 4	wild type			*not available *		
Patient 5	wild type			*not available *		
Patient 6	**mutated**	**PIK3CA AKT1**	**E545K E17K**	*not available *		
Patient 7	*not available*			**mutated **	**FBX4 PIK3CA KIT**	**G30N E545K S709F**
Patient 8	wild type			*not available *		
Patient 9	**mutated**	**TP53**	**R273H**	wild type		
Patient 10	wild type			**mutated **	**AKT1**	**E17K**
Patient 11	wild type			wild type		
Patient 12	wild type			*not available *		
Patient 13	wild type			**mutated **	**AKT1**	**E17K**
Patient 14	wild type			*not available *		
Patient 15	**mutated**	**KIT**	**G565R**	*not available *		
Patient 16	wild type			wild type		
Patient 17	*not available*			wild type		
Patient 18	**mutated**	**FBXW7**	**R479Q**	**mutated **	**MAP2K1**	**D67N**
Patient 19	wild type			wild type		
Patient 20	**mutated**	**CTNNB1**	**S45F**	wild type		
Patient 21	*not available*			wild type		
Patient 22	*not available*			**mutated **	**FBXW7**	**R465C**
Patient 23	wild type			*not available *		
Patient 24	*not available*			wild type		
Patient 25	wild type			*not available *		

**Table 3:** Correlation between mutations detected in primary tumor or relapse tissue and PFS on everolimus and exemestane.

	**Mutations in primary tumor or relapse tissue **	**PFS (months) **
Patient 1	wild type	3
Patient 2	**mutated (BRAF)**	6
Patient 3	wild type	4
Patient 4	wild type	5
Patient 5	wild type	2
Patient 6	**mutated (PIK3CA; AKT1)**	15
Patient 7	**mutated (FBX4; PIK3CA; KIT)**	3
Patient 8	wild type	17
Patient 9	**mutated (TP53)**	1
Patient 10	**mutated (AKT1)**	3
Patient 11	wild type	5
Patient 12	wild type	9
Patient 13	**mutated (AKT1)**	2
Patient 14	wild type	9
Patient 15	**mutated (KIT)**	3
Patient 16	wild type	8
Patient 17	wild type	13
Patient 18	**mutated (MAP2K1; FBXW7)**	6
Patient 19	wild type	5
Patient 20	**mutated (CTNNB1)**	8
Patient 21	wild type	12
Patient 22	**mutated (FBXW7)**	5
Patient 23	wild type	5
Patient 24	wild type	13
Patient 25	wild type	2

## Data Availability

The data regarding RNA extraction/concentration/purity are available from the corresponding author upon request.
